# Multiparametric Flow Cytometry Using Near-Infrared Fluorescent Proteins Engineered from Bacterial Phytochromes

**DOI:** 10.1371/journal.pone.0122342

**Published:** 2015-03-26

**Authors:** William G. Telford, Daria M. Shcherbakova, David Buschke, Teresa S. Hawley, Vladislav V. Verkhusha

**Affiliations:** 1 Experimental Transplantation and Immunology Branch, National Cancer Institute, National Institutes of Health, Bethesda, Maryland, United States of America; 2 Department of Anatomy and Structural Biology, Albert Einstein College of Medicine, Bronx, New York, United States of America; 3 Sony Biotechnology Incorporated, San Jose, California, United States of America; 4 Flow Cytometry Core Facility, George Washington University, Washington, DC, United States of America; 5 Department of Biochemistry and Developmental Biology, Faculty of Medicine, University of Helsinki, Helsinki, Finland; AntiCancer Inc., UNITED STATES

## Abstract

Engineering of fluorescent proteins (FPs) has followed a trend of achieving longer fluorescence wavelengths, with the ultimate goal of producing proteins with both excitation and emission in the near-infrared (NIR) region of the spectrum. Flow cytometers are now almost universally equipped with red lasers, and can now be equipped with NIR lasers as well. Most red-shifted FPs of the GFP-like family are maximally excited by orange lasers (590 to 610 nm) not commonly found on cytometers. This has changed with the development of the iRFP series of NIR FPs from the protein family of bacterial phytochromes. The shortest wavelength variants of this series, iRFP670 and iRFP682 showed maximal excitation with visible red lasers. The longer wavelength variants iRFP702, iRFP713 and iRFP720 could be optimally excited by NIR lasers ranging from 685 to 730 nm. Pairs of iRFPs could be detected simultaneously by using red and NIR lasers. Moreover, a novel spectral cytometry technique, which relies on spectral deconvolution rather than optical filters, allowed spectra of all five iRFPs to be analyzed simultaneously with no spectral overlap. Together, the combination of iRFPs with the advanced flow cytometry will allow to first image tissues expressing iRFPs deep in live animals and then quantify individual cell intensities and sort out the distinct primary cell subpopulations ex vivo.

## Introduction

Flow cytometry is a nearly ubiquitous technology in cell biology, particularly in the biomedical sciences. Flow cytometers permit the analysis of very large numbers of single cells, using lasers to excite cell-associated fluorescent probes, with filters and detectors to detect the often low levels of fluorescence associated with these markers. Genetically encoded fluorescent proteins (FPs) are important expressible markers in flow cytometry [**1,2**]. Since the development of enhanced mutants of a green FP (GFP) protein from *Aequorea victoria* jellyfish that can be excited with the blue-green 488 nm laser found on virtually every commercial instrument, a significant proportion of all flow cytometric analysis involves FP detection [**3**].

**Fig 1:**
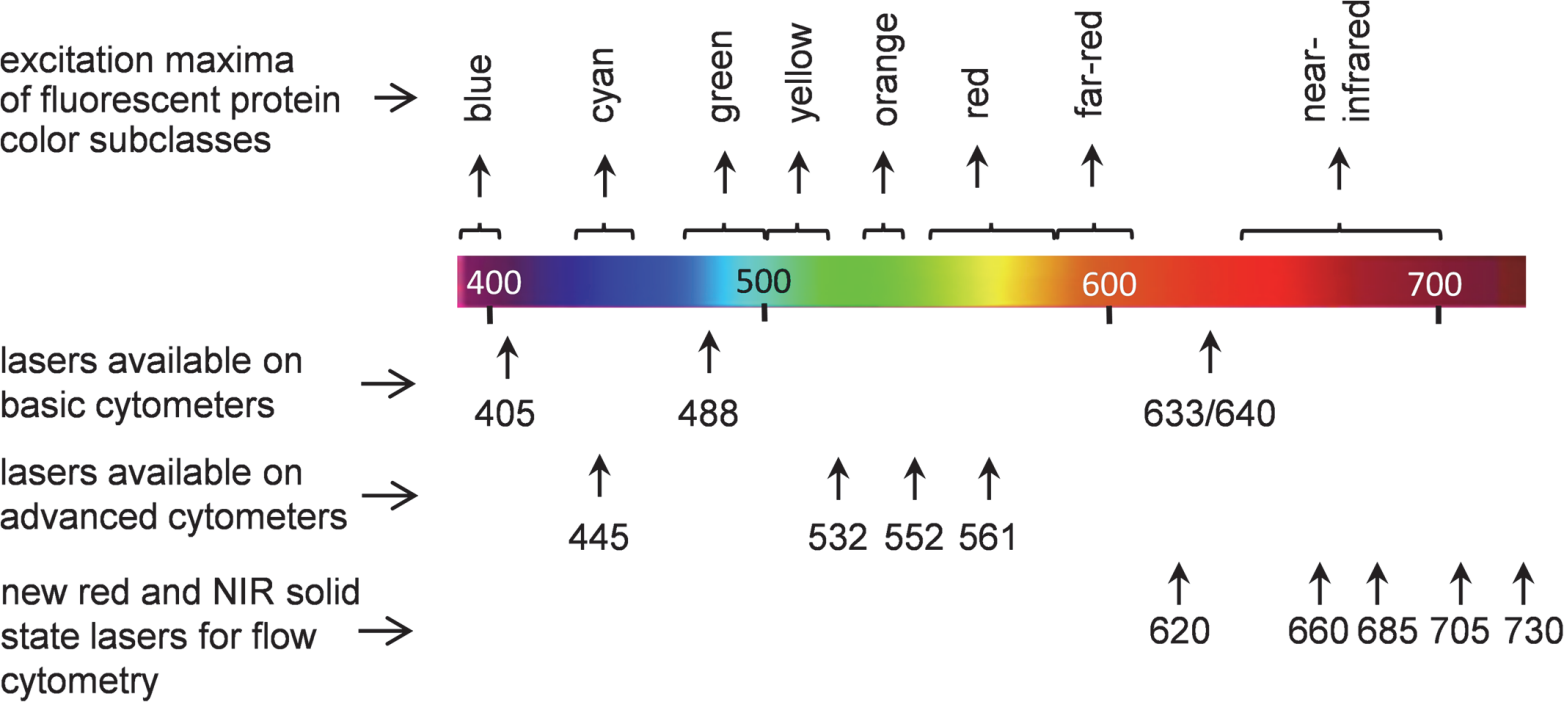
Typical array of laser wavelengths available on most flow cytometers. The array of the cytometry lasers is overlaid with the major groups of the GFP-like fluorescent proteins (from blue to far-red) and of the bacterial phytochrome based family of iRFP fluorescent proteins (near-infrared).

Basic flow cytometers are now typically equipped with two or three lasers in addition to the primary 488 nm, including red laser diodes (635 to 645 nm) and violet laser diodes (405 to 410 nm). These additional wavelengths give access to a larger array of fluorescent probes than a single laser wavelength alone. Red lasers in particular allow detection of many long red and NIR fluorescent probes, including phycobiliprotein probes like allophycocyanin (APC), and cyanine dyes and their many derivatives [**3,4,5**]. As with imaging, flow cytometric analysis with red lasers benefits from high sensitivity and low cellular autofluorescence. While useful for FP analysis, this group of lasers has its limitations for FPs. The GFP-derived enhanced FPs such as EGFP and EYFP can be efficiently excited at 488 nm, and ECFP can be excited using a violet source [**1,6**]. However, many of the more recently cloned red FPs (RFPs) of the GFP-like family from *Anthozoa* corals are optimally detected by many commercial instruments. Only the short-wavelength RFPs such as DsRed and dTomato can be excited somewhat at 488 nm, and with relatively low efficiency [**7,8**]. Longer-wavelength RFPs, like mCherry, require a green or yellow laser for excitation, with almost no excitation at 488 nm [**8**]. While these additional lasers are found on more advanced instrumentation, they are still absent from most simple commercial instruments. Fig. 1 shows the laser sources available on both basic and advanced instrumentation; for simpler instruments, there is a dearth of FPs that are accessible to their small array of laser wavelengths.

As the need for simultaneous analysis increasing numbers of FPs has grown, there has not been an increase in the FP selection available for conventional cytometers. The inexpensive red laser diodes available on most cytometers had until recently no role to play in FP excitation. There are long-wavelength RFPs with excitation maxima approaching “true red”, including mKate2, mNeptune, E2-Crimson, eqFP650, eqFP670, TagRFP657 and TagRFP675 [**9–15**]. However, the excitation maxima for these proteins ranged from 590 to 610 nm, well short of the typical red laser. Yellow to orange lasers (typically 592–594 nm) are used to excite these proteins, but these laser sources are found only on advanced instrumentation [**16,17**] Excitation of these proteins with red lasers is very inefficient and does not produce useful levels of fluorescent signal. There has been a tremendous need in flow cytometry for a RFP that excites well in the “true red” laser range, and emits in the far-red to near-infrared (NIR) range. Even more desirable would be multiple NIR FPs, which can be combined for measurement of several gene expression events and labelling of several cell populations.

The recently developed from bacterial phytochromes iRFP series of NIR FPs would appear to meet this requirement. This group of five proteins (iRFP670, iRFP682, iRFP702, iRFP713 and iRFP720) has excitation maxima ranging from 643 to 702 nm, and emission maxima ranging from 670 to 720 nm [**18,19**]. The NIR spectra of these proteins make them superior probes for deep-tissue imaging in mammals (Table 1). These proteins are already being used with great effect in whole-body fluorescence imaging traditionally performed with GFP-like FPs [**20,21**] While originally developed as probes for NIR imaging both in individual cells and whole organisms, their spectral properties make them ideal for flow cytometry. Their excitation and emission maxima strongly resemble fluorescent probes already in wide use in flow cytometry that rely on red laser excitation. In addition, the increasing incorporation of NIR lasers into cytometers can also allow optimal excitation and detection of the longest wavelength iRFPs.

**Table 1:** Comparison of near-infrared iRFPs engineered from bacterial phytochromes with several far-red FPs of the GFP-like family as probes for deep-tissue imaging.

**Fluorescent protein**	**Excitation maximum (nm)**	**Emission maximum (nm)**	**Molecular brightness** ^a^ ***vs*. iRFP713 (%)**	**Signal-to-background ratio at 7 mm depth in tissue *vs*. iRFP713** ^b^ **(%)**	**Signal-to-background ratio at 18 mm depth in tissue *vs*. iRFP713** ^b^ **(%)**
Far-red GFP-like FPs					
eqFP650	592	650	253	9	5
mNeptune	600	650	168	4	2
E2-Crimson	605	646	114	13	6
eqFP670	605	670	68	2	1
NIR FPs from bacterial phytochromes					
iRFP670	643	670	205	72	74
iRFP682	663	682	165	58	59
iRFP702	673	702	124	110	133
iRFP713	690	713	100	100	100
iRFP720	702	720	93	122	119

^a^Defined as a product of extinction coefficient and quantum yield. The molecular brightness values are from [**19**] for five iRFPs, [**10**] for mNeptune, [**11**] for E2-Crimson and [**13**] for eqFP650 and eqFP670.

^b^Data are from [**19**]. The signal-to-background ratios were measured for equal amounts of purified FPs imaged in a planar epifluorescence mode on an IVIS Spectrum instrument (PerkinElmer, Waltham, MA) inside a XFM-2 fluorescent phantom mouse (PerkinElmer) at the indicated depths from the mouse surface using the optimal filter channels for each FP.

In this study, the excitation and emission requirements for these probes were determined using a flow cytometry test bed and a range of red and NIR laser sources. The iRFPs were also combined in multicolor experiments to determine if two or more could be used simultaneously for measurement of multiple gene expression events. Finally, a recently developed spectral cytometer that uses prism-separated fluorescent signals and spectral deconvolution instead of filters was used to detect and separate the fluorescent signals from all five iRFPs simultaneously. Our studies indicated that the far-red excitation and NIR emission properties of the iRFP series of FPs engineered from bacterial phytochromes make them ideally suited for a wide range of flow cytometry applications.

## Materials and Methods

### Expression of iRFPs in bacterial cells

The pBAD-His/B plasmids containing genes of individual iRFP proteins under arabinose inducible promoter were transfected in LMG194 bacterial cells (Life Technologies, Carlsbad, CA) containing the pWA23h plasmid [**19,22**] encoding heme oxygenase for biliverdin production under rhamnose inducible promoter. Transfected *E*. *coli* bacteria were plated on Petri dishes with ampicillin, kanamycin, arabinose and rhamnose and grown for 24 hours at 37°C. The plated *E*. *coli* cells were scraped, re-suspended in a standard phosphate buffered saline, filtered to remove clumps and produce single-cell suspension, and fixed in 2% paraformaldehyde. The iRFP bacterial samples were analyzed by flow cytometry individually, and the data were merged.

### Traditional flow cytometry

All bacterial cell samples were analyzed on a LSRII quartz cuvette multi-laser flow cytometer (BD Biosciences, San Jose, CA) equipped with the following interchangeable red or NIR laser sources: (1) a diode pumped solid state 620 nm fiber laser (MPB Communications, Quebec, Canada), (2) a HeNe 633 nm red laser (JDS Uniphase, Germantown, MD), (3) a 637 nm red laser diode (Coherent, Carlsbad, CA), (4) a 660 nm red laser diode (Power Technology, Alexander, AR), (5) a 685 nm NIR laser diode (Power Technology), (6) a 705 nm NIR laser diode (Power Technology), and (7) a 730 nm NIR laser diode (Power Technology). All lasers used beam expansion optics (620 and 633 nm) or anamorphic optics (660, 685, 705 and 730 nm) to achieve circular beams (M < 1.3) with a diameter of 0.7 mm ±0.1 mm at a 1 meter focal length. All lasers were mounted sequentially on the cytometer, with light collection optics, fiber optics, PMTs, filters and dichroics used for each laser source. All lasers were attenuated to 20 mW power level at the flow cytometer flow cell for comparison purposes. For some experiments, both a red laser (620 nm) and a NIR laser were installed simultaneously, and aligned to spatially separated detectors for two-laser sample excitation. The bandpass filters used for FP detection were all manufactured by Semrock (Rochester, NY) unless otherwise noted and are designated in the text by their middle transmission wavelength followed by their window size (i.e. 740/13 nm). The photomultiplier tube (PMT) detectors on the LSR II were Hamamatsu 3896 multialkali PMTs with specifications for extended long red sensitivity up to 800 nm.

### Data analysis

All data was acquired using FACSDiVa data acquisition software (BD Biosciences, San Jose, CA) and analyzed using FlowJo version 7.6.5 (FlowJo LLC, Ashland, OR). The level of FP fluorescence was expressed as a staining index (SI), using the median and slope distributions of labeled and background cell or bead populations to calculate a value proportional to the level of fluorochrome fluorescence, as described previously [**23**]. Fluorescent compensation was calculated in FlowJo using the software package’s compensation algorithm.

### Spectral flow cytometry

The individual iRFP cell samples were analyzed on a SP6800 spectral cytometer (Sony Biotechnology, San Jose, CA,) equipped with a 488 nm laser emitting at 50 mW and a 637 nm red laser diode emitting at 60 mW. This instrument was equipped with a 32-channel PMT for spectral analysis over a 300 nm range. Spectral deconvolution and display as cytometric data was carried out using SP6800 data analysis software.

## Results

**Fig 2:**
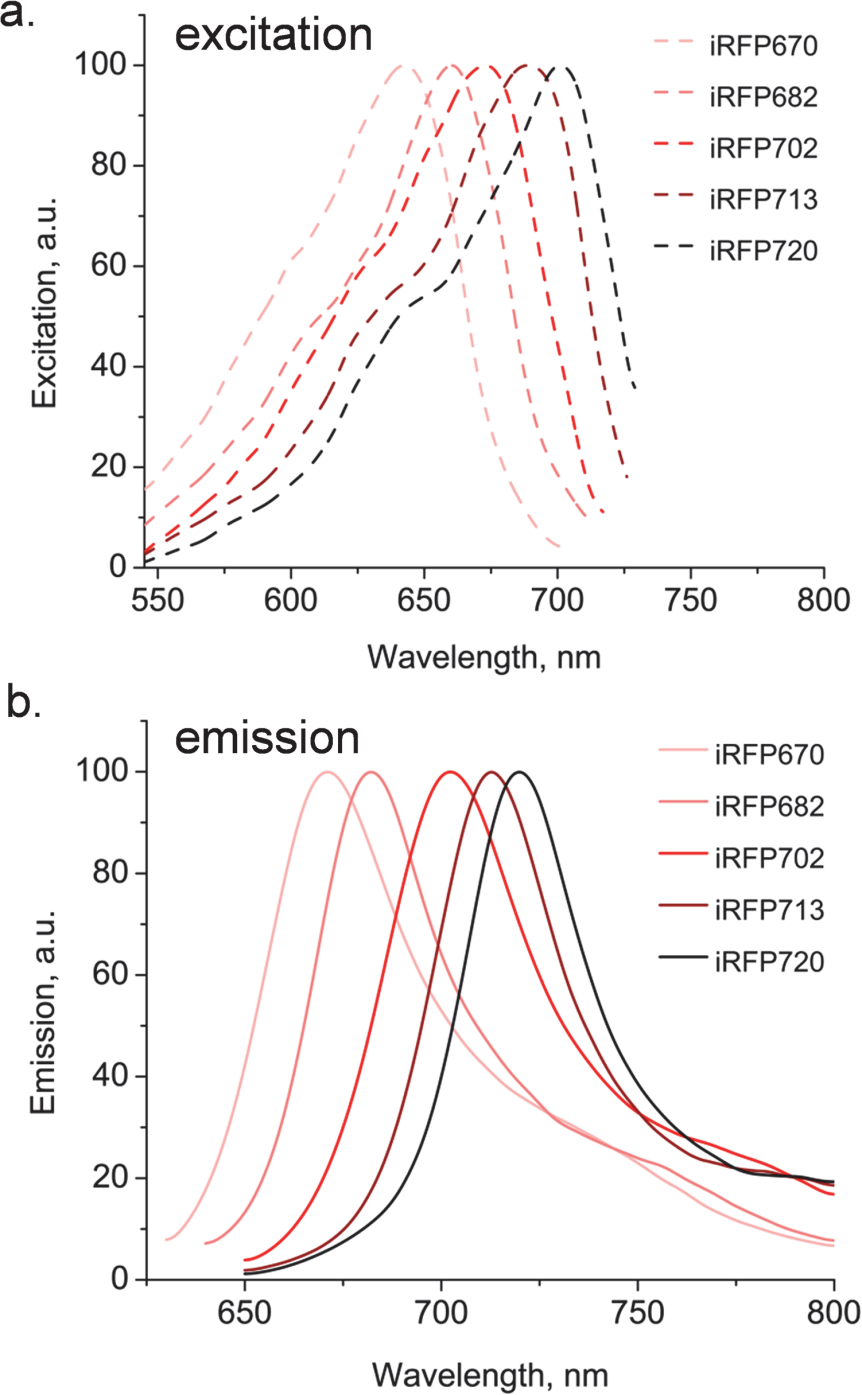
Fluorescence spectra for five purified iRFP proteins. Fluorescence excitation (**a**) and emission (**b**) spectra are shown for iRFP670, iRFP682, iRFP702, iRFP713 and iRFP720 fluorescent proteins.

**Fig 3:**
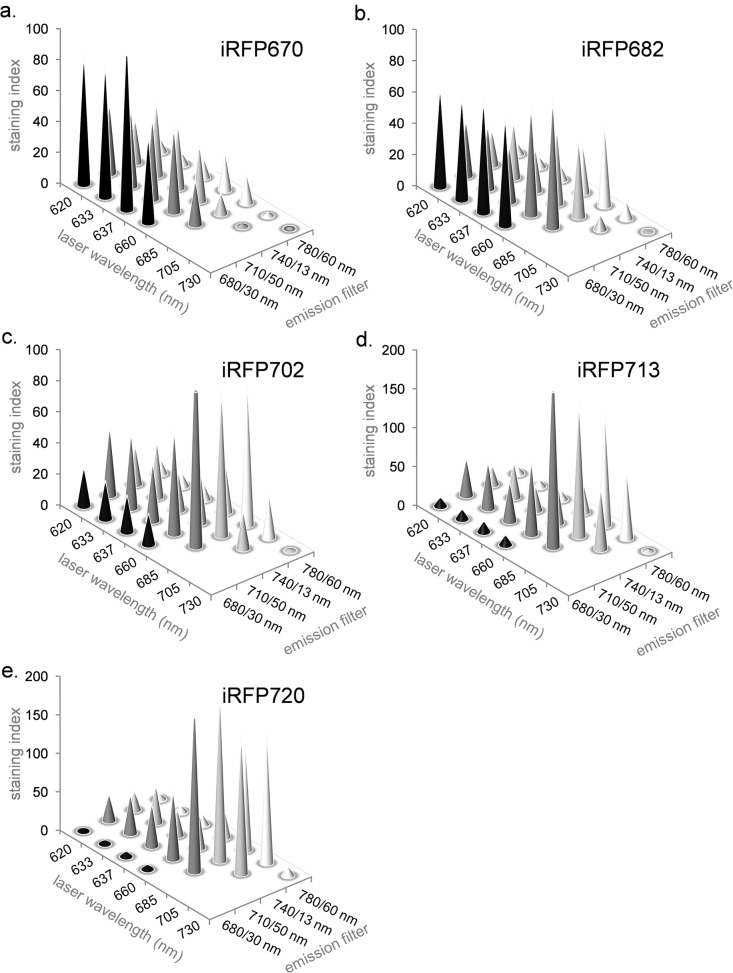
Fluorescence intensity of iRFP expressing cells with varying laser wavelengths and detection filters. The intensity of iRFP670 (**a**), iRFP682 (**b**), iRFP702 (**c**), iRFP713 (**d**) and iRFP720 (**e**) expressing samples are shown. Laser wavelength is plotted on the x-axis, and detection filter wavelength and window width is plotted on the y-axis. Fluorescence intensity on the z-axis is expressed as a staining index (SI), a relative value proportional to sample fluorescence versus background as described in the Materials and Methods.

The fluorescence excitation and emission spectra for iRFP670, iRFP682, iRFP702, iRFP713 and iRFP720 are shown in Fig. 2. The excitation spectra for all five proteins predict that both red and NIR laser should excite them well, and the emission spectra indicate that the filters traditionally used for red-excited probes should similarly be useful for these proteins. This was tested in Fig. 3, where *E*. *coli* expressing all five iRFPs were analyzed on a BD LSR II cuvette cytometer using lasers ranging from a 620 nm “short” red to a 730 nm NIR source, with a series of bandpass filters with center wavelengths ranging from 660 to 780 nm (shown on the x- and y-axes respectively). The staining index (SI) shown on the z-axis is a relative value for FP signal. iRFP670 was optimally excited by the 620, 633 and 637 nm red laser sources, and detected optimally using a 680/30 nm filter. Similarly, iRFP682 was also excited well using red sources, although the shortest NIR lasers also worked well. It was also optimally detected through a 680/30 nm filter, although a longer 710/50 nm filter could also be used. iRFP702, iRFP713 and iRFP720 were not well-excited in the red, requiring a 685 or 705 nm laser source for best detection. Detection of these proteins was in the NIR range of 710 to 780 nm. A 730 nm laser source was too long for good excitation of any of iRFPs.

**Fig 4:**
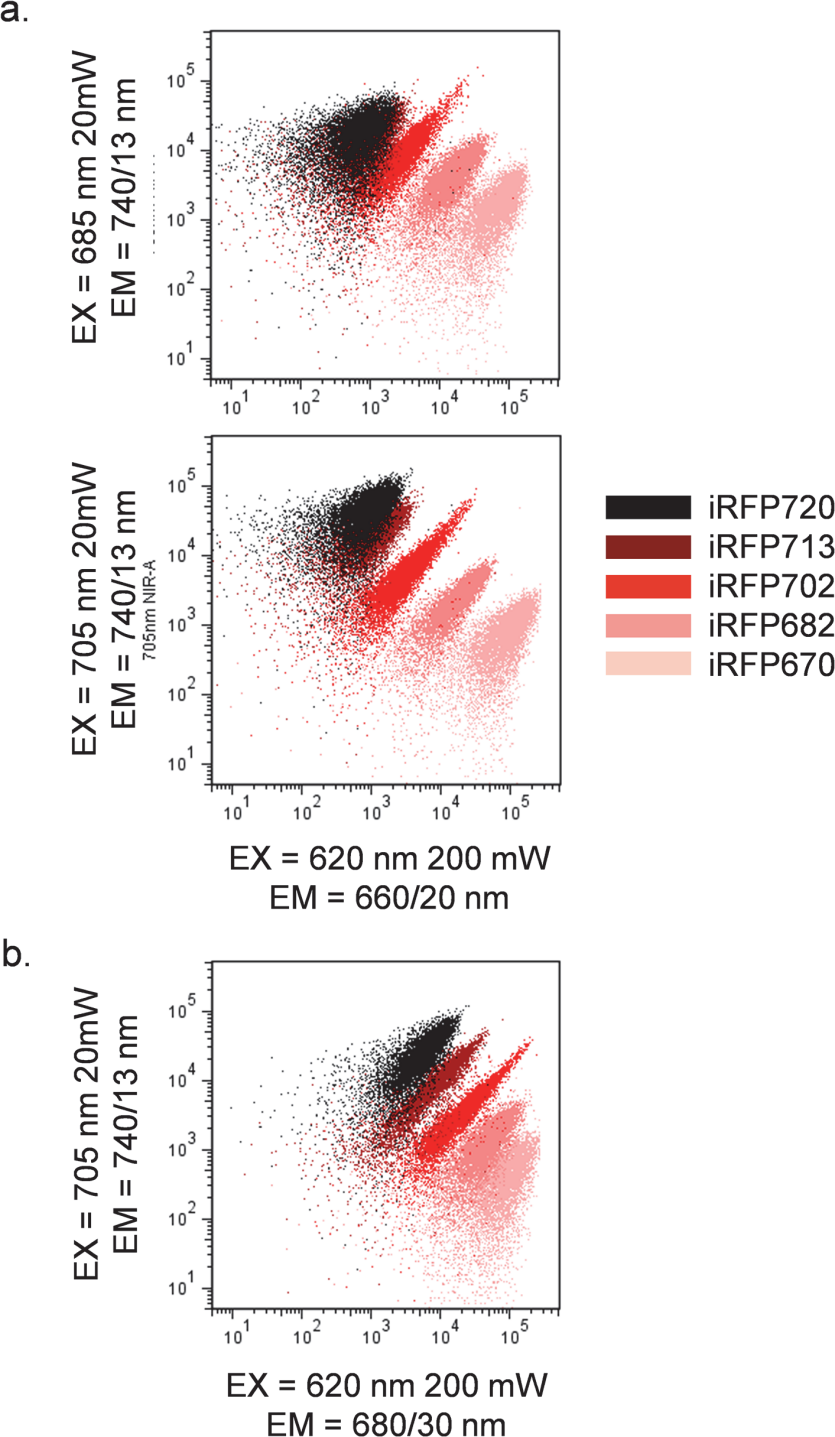
Fluorescence emission of iRFP expressing cells using dual red and NIR laser excitation. (**a**) Simultaneous excitation of all five iRFPs with spatially separated red 620 nm and either NIR 685 nm (top panel) or 705 nm (bottom panel) lasers, using detection using 660/20 nm and 740/13 nm filters respectively. Note that all FPs except iRFP713 and iRFP720 can be distinguished from each other. (**b**) Simultaneous excitation of all five iRFPs with red 620 nm and NIR 705 nm laser, with detection using 680/30 nm and 740/13 nm filters respectively. Note that all FPs can be distinguished from each other. No compensation or correction for fluorescence overlap was applied in this analysis.

Using the above excitation and emission data, it was then possible to test whether more than one iRFP could be detected simultaneously. The cytometer was configured with a red 620 nm laser to excite either iRFP670 or iRFP682, and a NIR 685 or 705 nm laser to excite iRFP702, iRFP713 or iRFP720. Fig. 4A shows the emission pattern for all five iRFPs using the 620 nm and either a 685 nm (top panel) or a 705 nm laser (bottom panel). All proteins except iRFP713 and iRFP720 could be distinguished from one another using this excitation/emission scheme. iRFP713 and iRFP720 were spectrally very similar with little discrimination using this laser and filter combination. iRFP713 and iRFP720 discrimination was improved by using a slightly longer red 680/30 nm filter in with 620 and 705 nm laser sources (Fig. 4B). Despite their spectral similarity, this combination of lasers and filters allowed discrimination of all five proteins.

**Fig 5:**
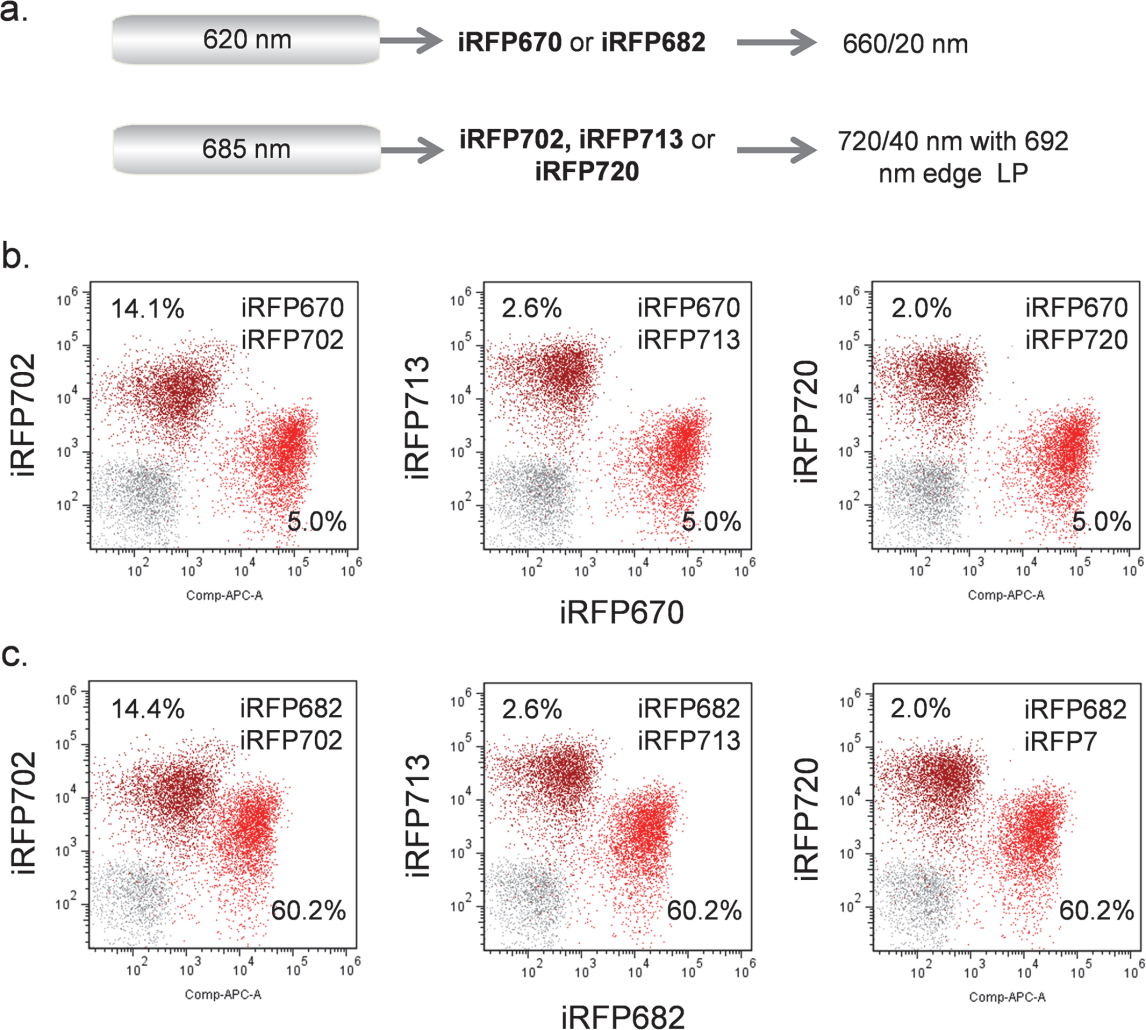
Simultaneous analysis of iRFP expressing samples using 620 nm and 685 nm lasers. (**a)** Laser and filter combinations. (**b**) Analysis of iRFP670 and iRFP702 (left), iRFP713 (middle) or iRFP720 (right) using the above lasers and filters. (**c**) Analysis of iRFP682 and iRFP702 (left), iRFP713 (middle) or iRFP720 (right) using the above lasers and filters. Data are compensated. The compensation values showing the subtraction of fluorescence overlap from each iRFP into the other is shown on each scatterplot.

While all iRFPs could be discriminated by flow cytometry, there was still considerable spectral overlap between the FPs. In flow cytometry, this overlap is usually subtracted by means of fluorescence compensation, a technique where unwanted fluorescence overlap is subtracted from adjacent fluorescent probes either electronically or using a software algorithm. This allows for groups of fluorochromes with close spectral properties to be used simultaneously for multiparametric analysis. Even with careful filter selection to minimize spectral overlap, some fluorescence compensation is usually required for multiple fluorochrome usage. In Fig. 5, the dual laser configuration described above with 685 nm as the NIR source was tested using either iRFP670 or iRF682 with red excitation, and iRFP702, iRFP713 or iRFP720 with NIR excitation (optical layout shown in Fig. 5A). The spectral compensation or spillover correction values are indicated as percentages on each scatterplot along with the actual fluorescence data. For all indicated combinations, the pairs of iRFPs could be used together with reasonable levels of fluorescence compensation (less than 100%).

**Fig 6:**
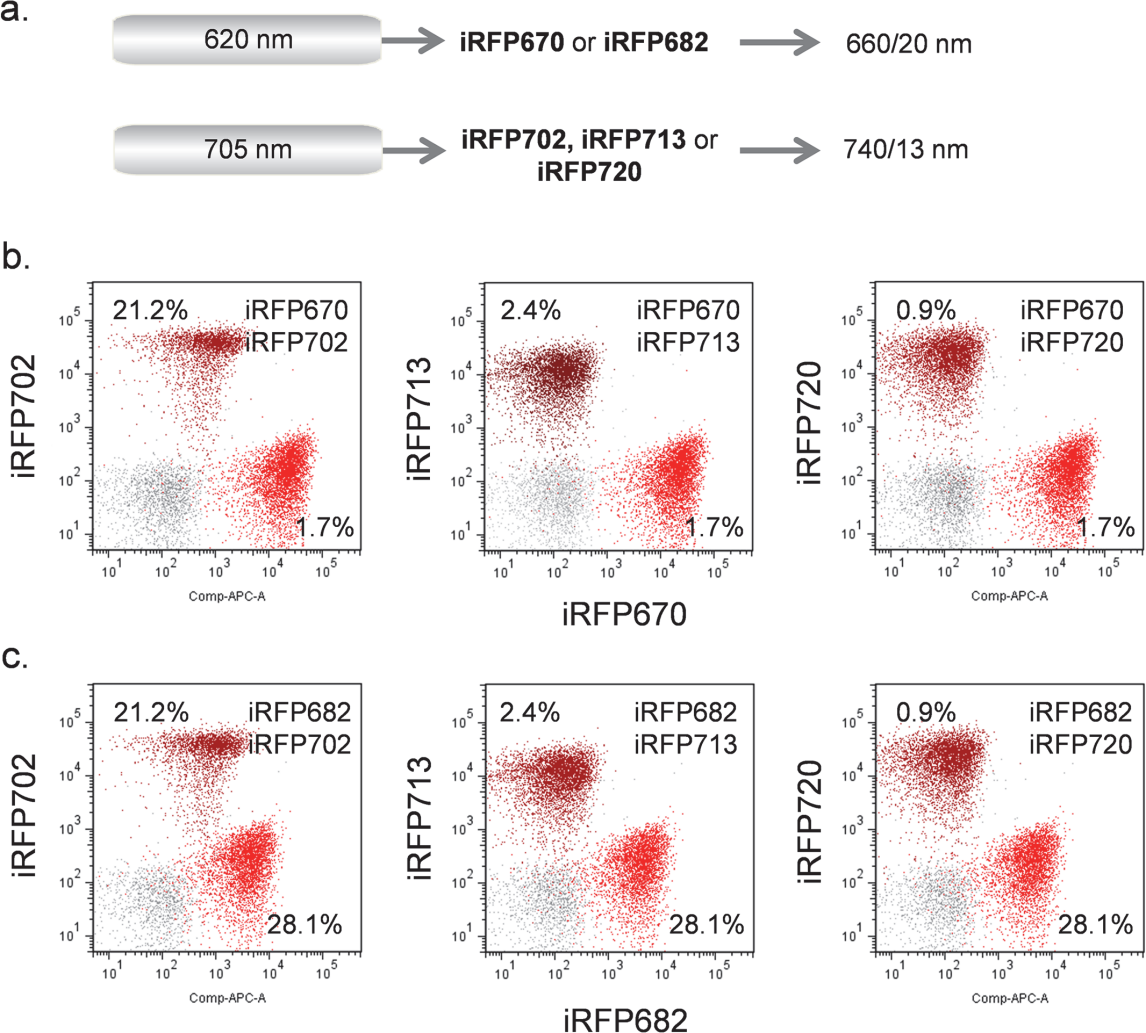
Simultaneous analysis of iRFP expressing samples using 620 nm and 705 nm lasers. (**a**) Laser and filter combinations. (**b**) Analysis of iRFP670 and iRFP702 (left), iRFP713 (middle) or iRFP720 (right) using the above lasers and filters. (**c**) Analysis of iRFP682 and iRFP702 (left), iRFP713 (middle) or iRFP720 (right) using the above lasers and filters. Data are compensated. The compensation values showing the subtraction of fluorescence overlap from each iRFP into the other is shown on each scatterplot.

Similar results were obtained in Fig. 6 by substituting the NIR 705 nm laser for the 685 nm. With this longer laser wavelength, a longer 740/13 nm filter was used instead of the 720/40 nm filter used with the 685 nm laser. This longer wavelength filter reduced the required fluorescence compensation without significantly reducing sensitivity. However, iRFP670 and iRFP682 were too close spectrally and could not be practically used together, despite their spectral difference shown in Fig. 4B; the required fluorescence compensation was too high (greater than 200%). Similarly IRFP702, iRFP713 and iRFP720 could also not be used together on a practical basis to due to their spectral similarity.

**Fig 7:**
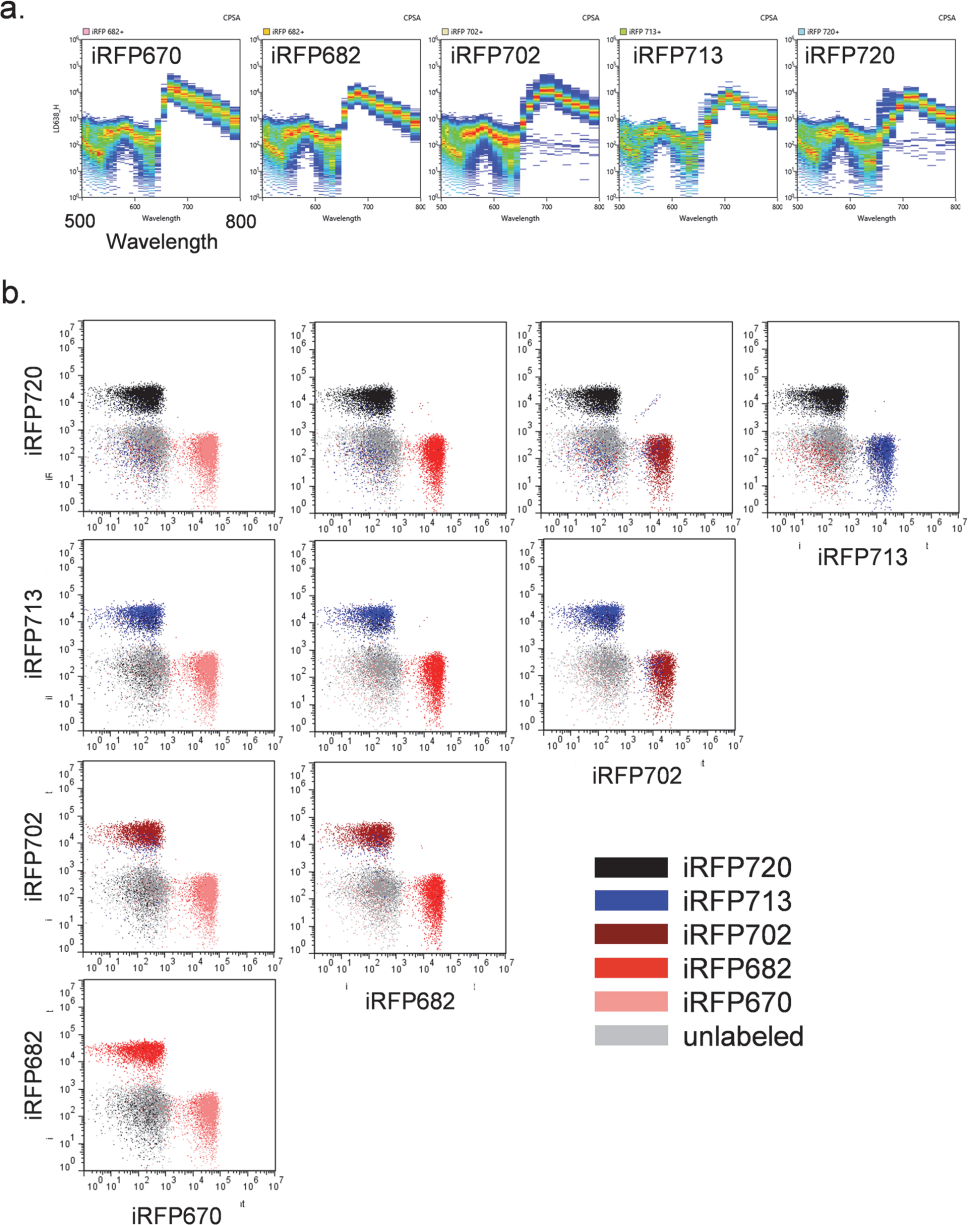
Spectral flow cytometry analysis of iRFP expressing cells. (**a**) Individual spectra for each iRFP using Sonly SP6800 spectral cytometer. (**b**) Analysis of all five iRFPs with data derived from spectral deconvolution of individual FP data.

While practical discrimination of more than two iRFPs was not possible by traditional flow cytometry, analysis by spectral cytometry was able to provide better iRFP signal separation. Spectral cytometry is a relatively new technology that uses an array of prisms to separate fluorescence signals into a spectral signature, with the resulting spectrum analyzed using a multichannel PMT. By collecting spectra on each of the fluorescent probes used in an experiment, it can use spectral deconvolution rather than filters and compensation to separate fluorochrome signals with similar spectral properties, and produce data similar to traditional flow cytometry. Fig. 7A shows the individual emission spectra for each iRFP as analyzed by a Sony SP6800 spectral flow cytometer, with excitation of the cells with a 638 nm red diode laser. These spectra were collected from the same bacterial cells used above; the resulting fluorescence data for all five iRFPs is shown in Fig. 7B. Spectral flow cytometry allows the detection and discrimination of all five iRFPs, even ones like iRFP713 and iRFP720 with extremely close spectral properties. This new form of flow cytometry will be very useful for analysis situations like this one, where fluorescent probes with similar spectral properties can be discriminated from one another.

## Discussion

All five iRFPs [**18,19**] proved to be useful genetically encoded NIR probes for flow cytometry. In contrast to most far-red FPs of the GFP-like family of proteins [**9–14**], iRFP670 and iRFP682 could be optimally excited by the red HeNe and laser diodes traditionally found on most flow cytometers. iRFP702, iRFP713 and iRFP720 required a NIR laser source, but these are now available on more advanced instrumentation. By combining red and NIR laser sources, pairs of either iRFP670 or iRFP682, and iRFP702, iRFP713 or iRFP720 could be analyzed together with relatively low levels of fluorescence compensation or spillover subtraction. More than two iRFPs were not spectrally practical by traditional flow cytometry, but all five iRFPs could be spectrally distinguished using newer spectral flow cytometry, giving the potential for analyzing many gene expression events and several labeled cell populations simultaneously.

The importance of NIR FPs for flow cytometry cannot be overemphasized. Although EGFP, EYFP, ECFP and their more recent variants were and remain very useful for flow cytometry, they have remained the upper limit for many simple cytometers possessing only blue-green, red and violet laser sources. All but the shortest wavelength RFPs are not well excited at 488 nm, and even short-wavelength RFPs, like DsRed and dTomato, only exhibit partial excitation in the blue-green. Most of the long-wavelength RFPs require a green or yellow laser source, usually available only on more advanced and expensive systems. Until recently, even the most red-shifted RFPs of the GFP-like family of proteins were poorly excited by the red HeNe and diode lasers that are almost ubiquitous on flow cytometers.

In conclusion, the ability to use both conventional red lasers and more recently incorporated near infrared lasers to excite NIR FPs [**18,19**] tremendously expands the ability to do multiparametric FP analysis of multiple intracellular events even on simple cytometers. In addition to the more conventional use of these proteins in cell lines, the combination of these FPs and flow cytometry will allow investigators to first detect fluorescence levels and to image localization of tissues and organs expressing iRFPs in a live animal *in vivo* and then quantify individual cell intensities and sort out the distinct primary cell subpopulations *ex vivo*. Moreover, other recently engineered iRFP variants, such as photoactivatable PAiRFPs [**22**] and an iSplit reporter of protein-protein interaction [**24**], should provide additional possibilities to flow cytometric assays of cells photolabeled by the PAiRFP photoactivation or visualized by iSplit as a result of the protein complex formation.
